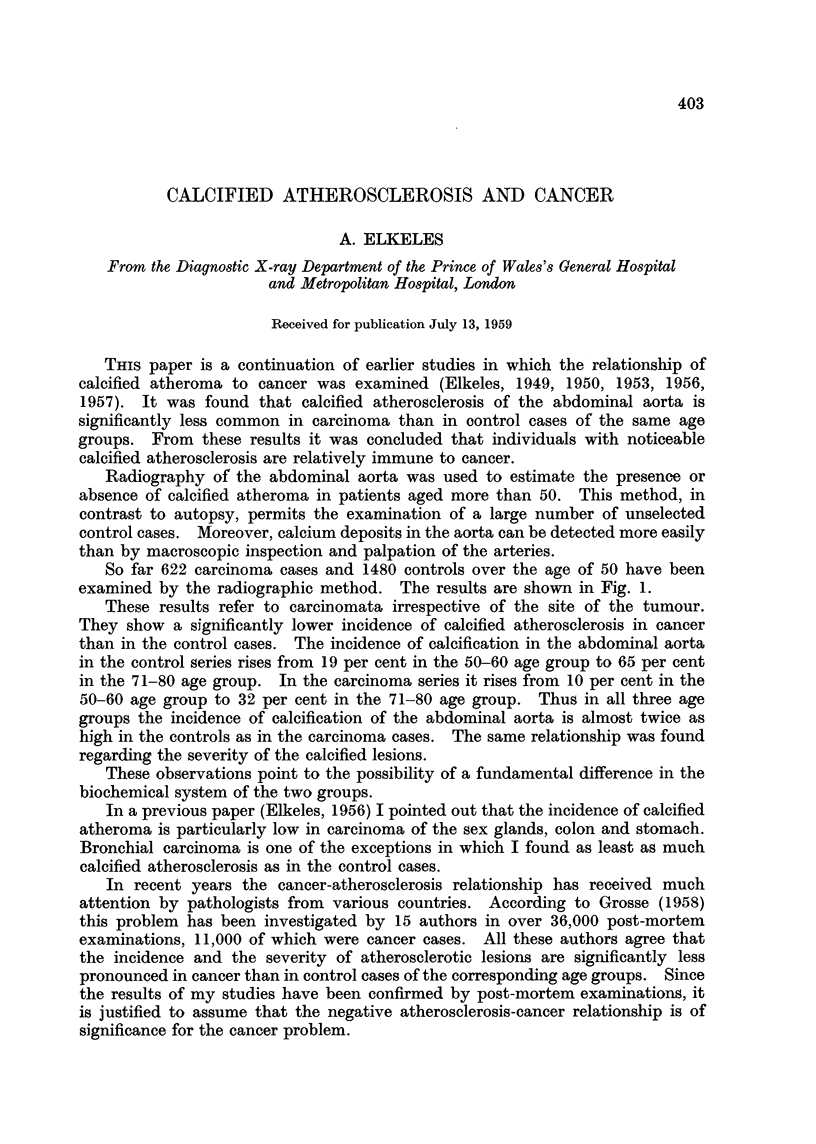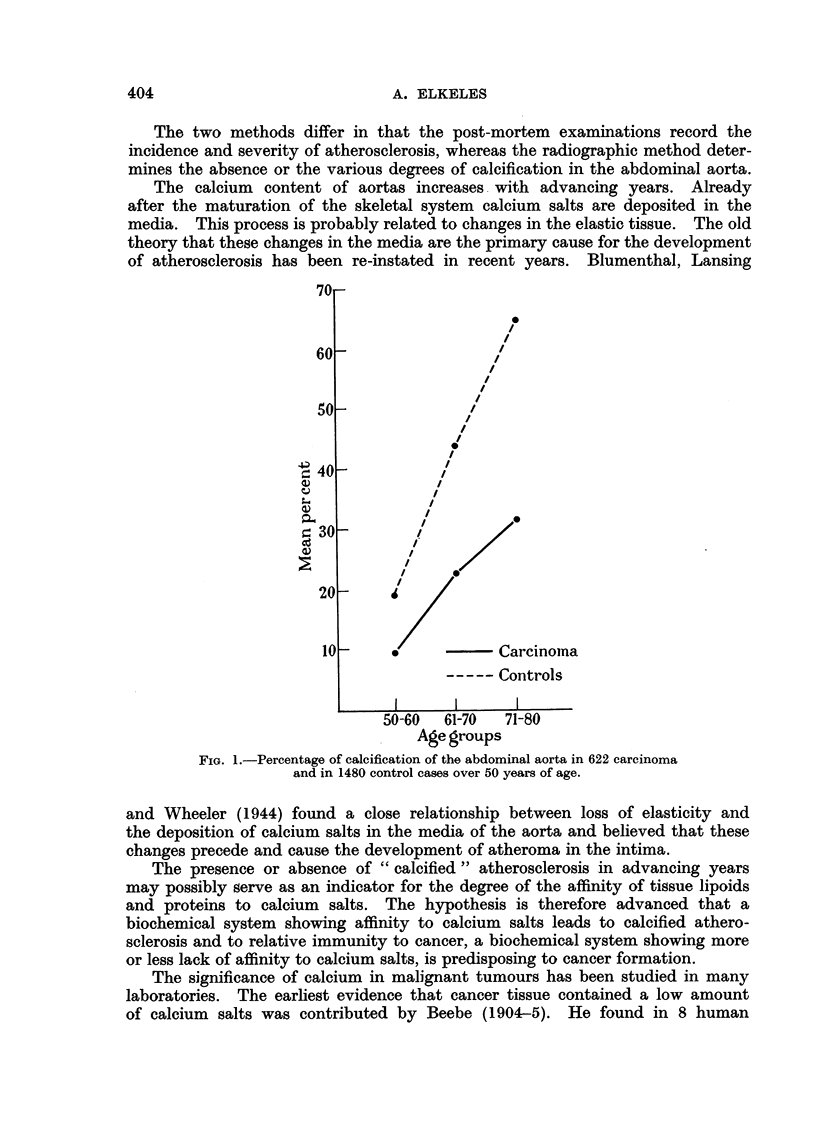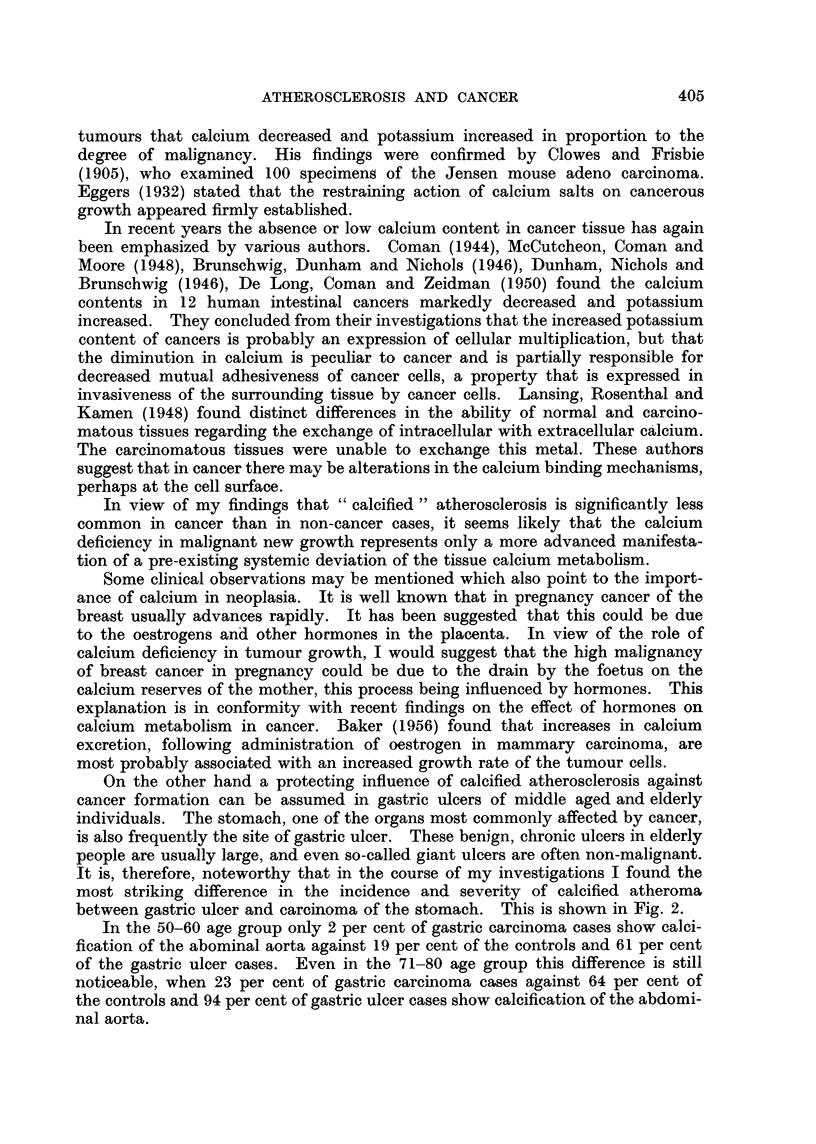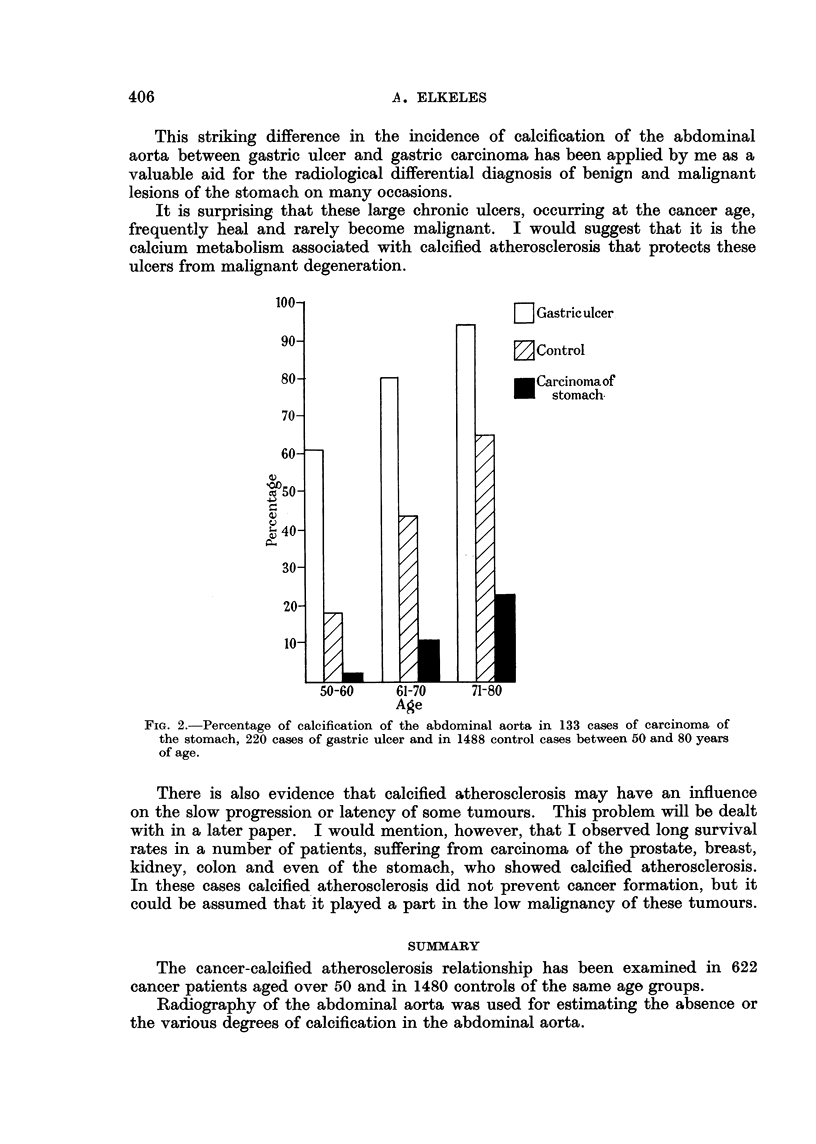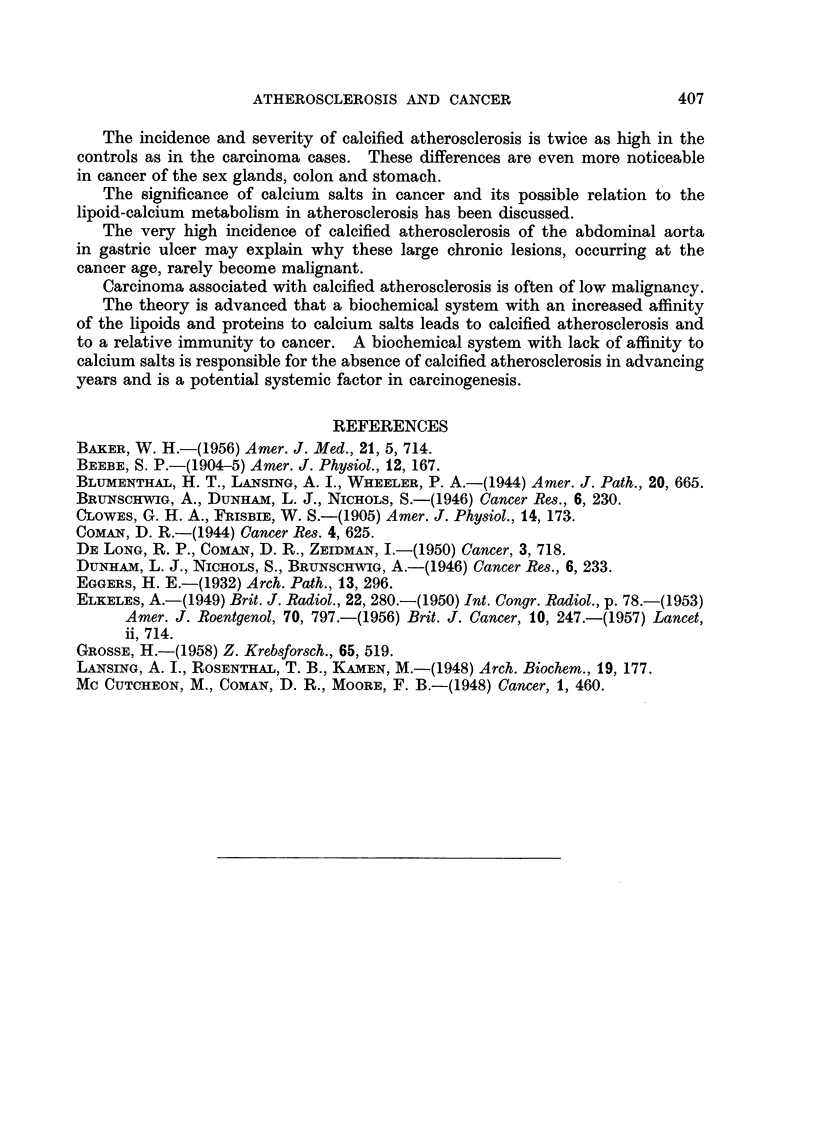# Calcified Atherosclerosis and Cancer

**DOI:** 10.1038/bjc.1959.46

**Published:** 1959-09

**Authors:** A. Elkeles


### CALCIFIED ATHEROSCLEROSIS AND CANCER

A. ELKELES

*From the Diagnostic X-ray Department of the Prince of Wales's General Hospital and Metropolitan Hospital, London*

Received for publication July 13, 1959

THIS paper is a continuation of earlier studies in which the relationship of calcified atheroma to cancer was examined (Elkeles, 1949, 1950, 1953, 1956, 1957). It was found that calcified atherosclerosis of the abdominal aorta is significantly less common in carcinoma than in control cases of the same age groups. From these results it was concluded that individuals with noticeable calcified atherosclerosis are relatively immune to cancer.

Radiography of the abdominal aorta was used to estimate the presence or absence of calcified atheroma in patients aged more than 50. This method, in contrast to autopsy, permits the examination of a large number of unselected control cases. Moreover, calcium deposits in the aorta can be detected more easily than by macroscopic inspection and palpation of the arteries.

So far 622 carcinoma cases and 1480 controls over the age of 50 have been examined by the radiographic method. The results are shown in Fig. 1.

These results refer to carcinomata irrespective of the site of the tumour. They show a significantly lower incidence of calcified atherosclerosis in cancer than in the control cases. The incidence of calcification in the abdominal aorta in the control series rises from 19 per cent in the 50–60 age group to 65 per cent in the 71–80 age group. In the carcinoma series it rises from 10 per cent in the 50–60 age group to 32 per cent in the 71–80 age group. Thus in all three age groups the incidence of calcification of the abdominal aorta is almost twice as high in the controls as in the carcinoma cases. The same relationship was found regarding the severity of the calcified lesions.

These observations point to the possibility of a fundamental difference in the biochemical system of the two groups.

In a previous paper (Elkeles, 1956) I pointed out that the incidence of calcified atheroma is particularly low in carcinoma of the sex glands, colon and stomach. Bronchial carcinoma is one of the exceptions in which I found as least as much calcified atherosclerosis as in the control cases.

In recent years the cancer-atherosclerosis relationship has received much attention by pathologists from various countries. According to Grosse (1958) this problem has been investigated by 15 authors in over 36,000 post-mortem examinations, 11,000 of which were cancer cases. All these authors agree that the incidence and the severity of atherosclerotic lesions are significantly less pronounced in cancer than in control cases of the corresponding age groups. Since the results of my studies have been confirmed by post-mortem examinations, it is justified to assume that the negative atherosclerosis-cancer relationship is of significance for the cancer problem.

The two methods differ in that the post-mortem examinations record the incidence and severity of atherosclerosis, whereas the radiographic method determines the absence or the various degrees of calcification in the abdominal aorta.

The calcium content of aortas increases with advancing years. Already after the maturation of the skeletal system calcium salts are deposited in the media. This process is probably related to changes in the elastic tissue. The old theory that these changes in the media are the primary cause for the development of atherosclerosis has been re-instated in recent years. Blumenthal, Lansing

FIG. 1.—Percentage of calcification of the abdominal aorta in 622 carcinoma and in 1480 control cases over 50 years of age.

and Wheeler (1944) found a close relationship between loss of elasticity and the deposition of calcium salts in the media of the aorta and believed that these changes precede and cause the development of atheroma in the intima.

The presence or absence of " calcified " atherosclerosis in advancing years may possibly serve as an indicator for the degree of the affinity of tissue lipoids and proteins to calcium salts. The hypothesis is therefore advanced that a biochemical system showing affinity to calcium salts leads to calcified atherosclerosis and to relative immunity to cancer, a biochemical system showing more or less lack of affinity to calcium salts, is predisposing to cancer formation.

The significance of calcium in malignant tumours has been studied in many laboratories. The earliest evidence that cancer tissue contained a low amount of calcium salts was contributed by Beebe (1904–5). He found in 8 human tumours that calcium decreased and potassium increased in proportion to the degree of malignancy. His findings were confirmed by Clowes and Frisbie (1905), who examined 100 specimens of the Jensen mouse adeno carcinoma. Eggers (1932) stated that the restraining action of calcium salts on cancerous growth appeared firmly established.

In recent years the absence or low calcium content in cancer tissue has again been emphasized by various authors. Coman (1944), McCutcheon, Coman and Moore (1948), Brunschwig, Dunham and Nichols (1946), Dunham, Nichols and Brunschwig (1946), De Long, Coman and Zeidman (1950) found the calcium contents in 12 human intestinal cancers markedly decreased and potassium increased. They concluded from their investigations that the increased potassium content of cancers is probably an expression of cellular multiplication, but that the diminution in calcium is peculiar to cancer and is partially responsible for decreased mutual adhesiveness of cancer cells, a property that is expressed in invasiveness of the surrounding tissue by cancer cells. Lansing, Rosenthal and Kamen (1948) found distinct differences in the ability of normal and carcinomatous tissues regarding the exchange of intracellular with extracellular calcium. The carcinomatous tissues were unable to exchange this metal. These authors suggest that in cancer there may be alterations in the calcium binding mechanisms, perhaps at the cell surface.

In view of my findings that " calcified " atherosclerosis is significantly less common in cancer than in non-cancer cases, it seems likely that the calcium deficiency in malignant new growth represents only a more advanced manifestation of a pre-existing systemic deviation of the tissue calcium metabolism.

Some clinical observations may be mentioned which also point to the importance of calcium in neoplasia. It is well known that in pregnancy cancer of the breast usually advances rapidly. It has been suggested that this could be due to the oestrogens and other hormones in the placenta. In view of the role of calcium deficiency in tumour growth, I would suggest that the high malignancy of breast cancer in pregnancy could be due to the drain by the foetus on the calcium reserves of the mother, this process being influenced by hormones. This explanation is in conformity with recent findings on the effect of hormones on calcium metabolism in cancer. Baker (1956) found that increases in calcium excretion, following administration of oestrogen in mammary carcinoma, are most probably associated with an increased growth rate of the tumour cells.

On the other hand a protecting influence of calcified atherosclerosis against cancer formation can be assumed in gastric ulcers of middle aged and elderly individuals. The stomach, one of the organs most commonly affected by cancer, is also frequently the site of gastric ulcer. These benign, chronic ulcers in elderly people are usually large, and even so-called giant ulcers are often non-malignant. It is, therefore, noteworthy that in the course of my investigations I found the most striking difference in the incidence and severity of calcified atheroma between gastric ulcer and carcinoma of the stomach. This is shown in Fig. 2.

In the 50–60 age group only 2 per cent of gastric carcinoma cases show calcification of the abominal aorta against 19 per cent of the controls and 61 per cent of the gastric ulcer cases. Even in the 71–80 age group this difference is still noticeable, when 23 per cent of gastric carcinoma cases against 64 per cent of the controls and 94 per cent of gastric ulcer cases show calcification of the abdominal aorta.

This striking difference in the incidence of calcification of the abdominal aorta between gastric ulcer and gastric carcinoma has been applied by me as a valuable aid for the radiological differential diagnosis of benign and malignant lesions of the stomach on many occasions.

It is surprising that these large chronic ulcers, occurring at the cancer age, frequently heal and rarely become malignant. I would suggest that it is the calcium metabolism associated with calcified atherosclerosis that protects these ulcers from malignant degeneration.

FIG. 2.—Percentage of calcification of the abdominal aorta in 133 cases of carcinoma of the stomach, 220 cases of gastric ulcer and in 1488 control cases between 50 and 80 years of age.

There is also evidence that calcified atherosclerosis may have an influence on the slow progression or latency of some tumours. This problem will be dealt with in a later paper. I would mention, however, that I observed long survival rates in a number of patients, suffering from carcinoma of the prostate, breast, kidney, colon and even of the stomach, who showed calcified atherosclerosis. In these cases calcified atherosclerosis did not prevent cancer formation, but it could be assumed that it played a part in the low malignancy of these tumours.

### SUMMARY

The cancer-calcified atherosclerosis relationship has been examined in 622 cancer patients aged over 50 and in 1480 controls of the same age groups.

Radiography of the abdominal aorta was used for estimating the absence or the various degrees of calcification in the abdominal aorta.

The incidence and severity of calcified atherosclerosis is twice as high in the controls as in the carcinoma cases. These differences are even more noticeable in cancer of the sex glands, colon and stomach.

The significance of calcium salts in cancer and its possible relation to the lipoid-calcium metabolism in atherosclerosis has been discussed.

The very high incidence of calcified atherosclerosis of the abdominal aorta in gastric ulcer may explain why these large chronic lesions, occurring at the cancer age, rarely become malignant.

Carcinoma associated with calcified atherosclerosis is often of low malignancy.

The theory is advanced that a biochemical system with an increased affinity of the lipoids and proteins to calcium salts leads to calcified atherosclerosis and to a relative immunity to cancer. A biochemical system with lack of affinity to calcium salts is responsible for the absence of calcified atherosclerosis in advancing years and is a potential systemic factor in carcinogenesis.

### REFERENCES

Baker, W. H.—(1956) *Amer. J. Med.*, **21**, 5, 714.
Beebe, S. P.—(1904–5) *Amer. J. Physiol.*, **12**, 167.
Blumenthal, H. T., Lansing, A. I., Wheeler, P. A.—(1944) *Amer. J. Path.*, **20**, 665.
Brunschwig, A., Dunham, L. J., Nichols, S.—(1946) *Cancer Res.*, **6**, 230.
Clowes, G. H. A., Frisbie, W. S.—(1905) *Amer. J. Physiol.*, **14**, 173.
Coman, D. R.—(1944) *Cancer Res.* **4**, 625.
De Long, R. P., Coman, D. R., Zeidman, I.—(1950) *Cancer*, **3**, 718.
Dunham, L. J., Nichols, S., Brunschwig, A.—(1946) *Cancer Res.*, **6**, 233.
Eggers, H. E.—(1932) *Arch. Path.*, **13**, 296.
Elkeles, A.—(1949) *Brit. J. Radiol.*, **22**, 280.—(1950) *Int. Congr. Radiol.*, p. 78.—(1953) *Amer. J. Roentgenol*, **70**, 797.—(1956) *Brit. J. Cancer*, **10**, 247.—(1957) *Lancet*, ii, 714.
Grosse, H.—(1958) *Z. Krebsforsch.*, **65**, 519.
Lansing, A. I., Rosenthal, T. B., Kamen, M.—(1948) *Arch. Biochem.*, **19**, 177.
Mc Cutcheon, M., Coman, D. R., Moore, F. B.—(1948) *Cancer*, **1**, 460.